# Glucose-6-phosphate dehydrogenase deficiency presenting with rhabdomyolysis in a patient with coronavirus disease 2019 pneumonia: a case report

**DOI:** 10.1186/s13256-022-03322-w

**Published:** 2022-03-14

**Authors:** Regina Yu, Chien-Rong Chen, Darci Evans, Xin Qing, Moran Gotesman, Gangadarshni Chandramohan, Thomas Kallay, Henry J. Lin, Tiffany P. Pedigo

**Affiliations:** 1 Division of Pediatric Critical Care, Department of Pediatrics, Harbor-UCLA Medical Center, 1000 W Carson St, Building N-25, Box 491, Torrance, CA 90502 USA; 2 Department of Pediatrics, Harbor-UCLA Medical Center, Torrance, CA USA; 3 Department of Pathology, Harbor-UCLA Medical Center, Torrance, CA USA; 4 Division of Pediatric Hematology/Oncology, Department of Pediatrics, Harbor-UCLA Medical Center, Torrance, CA USA; 5 Division of Pediatric Nephrology, Department of Pediatrics, Harbor-UCLA Medical Center, Torrance, CA USA; 6 Division of Medical Genetics, Department of Pediatrics, Harbor-UCLA Medical Center, Torrance, CA USA

**Keywords:** Rhabdomyolysis, G6PD deficiency, Acute kidney injury, COVID-19, SARS-CoV-2, Methemoglobinemia, Case report

## Abstract

**Background:**

Glucose-6-phosphate dehydrogenase deficiency is a rarely recognized predisposing factor for rhabdomyolysis. Rhabdomyolysis with coronavirus disease 2019 has been increasingly seen during the pandemic. We report the uncommon occurrence of coronavirus disease 2019 pneumonia, severe rhabdomyolysis, and acute renal failure in the setting of glucose-6-phosphate dehydrogenase deficiency.

**Case presentation:**

A 19-year-old African American male presented with myalgias, diaphoresis, and dark urine. Testing for severe acute respiratory syndrome coronavirus 2 was positive. He had severe rhabdomyolysis with creatine kinase levels up to 346,695 U/L. He was oliguric and eventually required hemodialysis. Progressive hypoxemia, methemoglobinemia, and hemolytic anemia occurred following one dose of rasburicase for hyperuricemia. Glucose-6-phosphate dehydrogenase deficiency was diagnosed. Full recovery followed a single volume exchange transfusion and simple packed red blood cell transfusions.

**Conclusions:**

Glucose-6-phosphate dehydrogenase deficiency may predispose individuals to rhabdomyolysis due to severe acute respiratory syndrome coronavirus 2, presumably due to altered host responses to viral oxidative stress. Early screening for glucose-6-phosphate dehydrogenase deficiency can be useful for management of patients with rhabdomyolysis.

## Background

Glucose-6-phosphate dehydrogenase (G6PD) deficiency is a potentially dangerous enzymopathy that affects more than 400 million people worldwide. The condition is X-linked and more common among African, Asian, Mediterranean, and Middle Eastern males [1]. G6PD is normally present in all cells for production of nicotinamide adenine dinucleotide phosphate (NADPH) [2]. NADPH is the reducing agent for biosynthesis of fatty acids, cholesterol, deoxyribose, and other compounds. At the same time, NADPH defends cells against oxidative stress. Oxidative damage in red blood cells can lead to hemolysis [3]. Furthermore, when hemoglobin is oxidized to methemoglobin, its ability to carry oxygen is impaired [4].

Glucose-6-phosphate dehydrogenase deficiency also predisposes individuals to rhabdomyolysis from oxidative stress in skeletal muscle [5]. Although rare in individuals with G6PD deficiency, rhabdomyolysis can lead to life-threatening cardiac arrhythmias or acute renal failure [6, 7]. Rhabdomyolysis has also been observed with SARS-CoV-2 infection, though the exact mechanism of muscle damage has not been established [8].

We report an African American male with rhabdomyolysis, acute renal failure, and COVID-19 pneumonia. He developed hypoxemia and methemoglobinemia after a single dose of rasburicase for hyperuricemia, leading to a diagnosis of G6PD deficiency. The patient illustrates an unusual presentation of G6PD deficiency with severe rhabdomyolysis and SARS-CoV-2 infection.

## Case presentation

A 19-year-old African American male was admitted to the hospital with 2 days of myalgias, decreased appetite, and diaphoresis followed by 1 day of scant, dark urine. He had been in good health, except for obesity, obstructive sleep apnea, and asthma in the past. He had no respiratory symptoms or history of intense physical activity or trauma. He used marijuana by vaping a few days before admission but denied other drug use. There was a history of myocardial infarction at young ages in multiple relatives.

Vital signs were normal except for a heart rate of 113. He appeared comfortable but diaphoretic. Physical examination was otherwise unremarkable, including soft limb compartments. Initial laboratory values were hemoglobin 160 g/L (normal 135–165 g/L), hematocrit 48.2% (normal 40–49%), MCV 84 fL (normal 82–97 fL), blood urea nitrogen 5.7 mmol/L (normal 2.9–7.1 mmol/L), creatinine 160.9 μmol/L (56.6–112.3 μmol/L), AST 1234 U/L (normal 15–41 U/L), ALT 123 U/L (normal 10–40 U/L), creatine kinase 346,695 U/L (normal 49–397 U/L), and uric acid 868 μmol/L (normal 285.5–517.5 μmol/L). Urinalysis showed “large” blood with 0–2 red blood cells per high-power field. SARS-CoV-2 testing was positive by polymerase chain reaction (PCR). Chest radiograph and renal ultrasound imaging were normal.

The patient was admitted to the pediatric intensive care unit for management of rhabdomyolysis and acute kidney injury. Intravenous fluids and sodium bicarbonate were used to lessen the risk of further renal injury. One dose of rasburicase was given intravenously (30 mg), as treatment for hyperuricemia due to rhabdomyolysis [9]. He remained oliguric, and continuous renal replacement therapy was started. He became febrile (39.2 °C). Progressive hypoxemia developed over the next 24 hours despite supplemental oxygen via high-flow nasal cannula (HFNC). Helical computed tomography of the chest showed bilateral ground-glass opacities consistent with pneumonia but no evidence of pulmonary embolism. He was given empiric azithromycin and ceftriaxone for community-acquired pneumonia and remdesivir and dexamethasone, the only recommended therapies for SARS-CoV-2 pneumonia at that time.

**Fig. 1:**
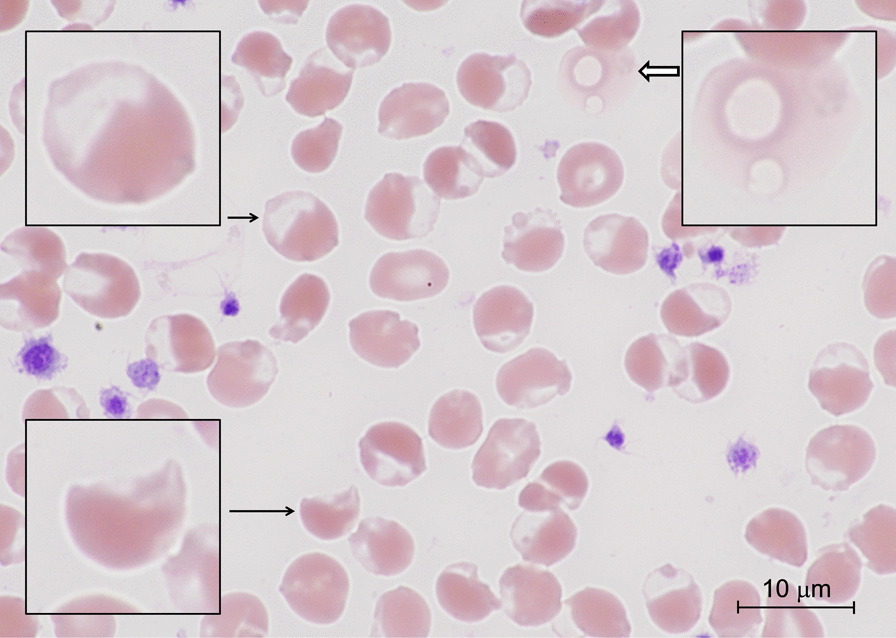
Peripheral blood smear. A peripheral blood smear showing occasional hemolyzed cells (thick arrow), many blister cells (short arrow), and a few bite cells (long arrow) (Wright–Giemsa stain)

On hospital day 2, the patient was intubated for hypoxemic respiratory failure. An arterial blood gas after intubation showed an oxygen saturation gap, with a PaO_2_ of 197 mmHg (26.3 kPa) but pulse oximetry readings of 82–86% saturation. The methemoglobin level was 11.1% by co-oximetry. Bite cells and blister cells on peripheral blood smears suggested oxidative stress and hemolysis (Fig. 1). A qualitative test showed markedly reduced G6PD enzyme activity.

**Fig. 2:**
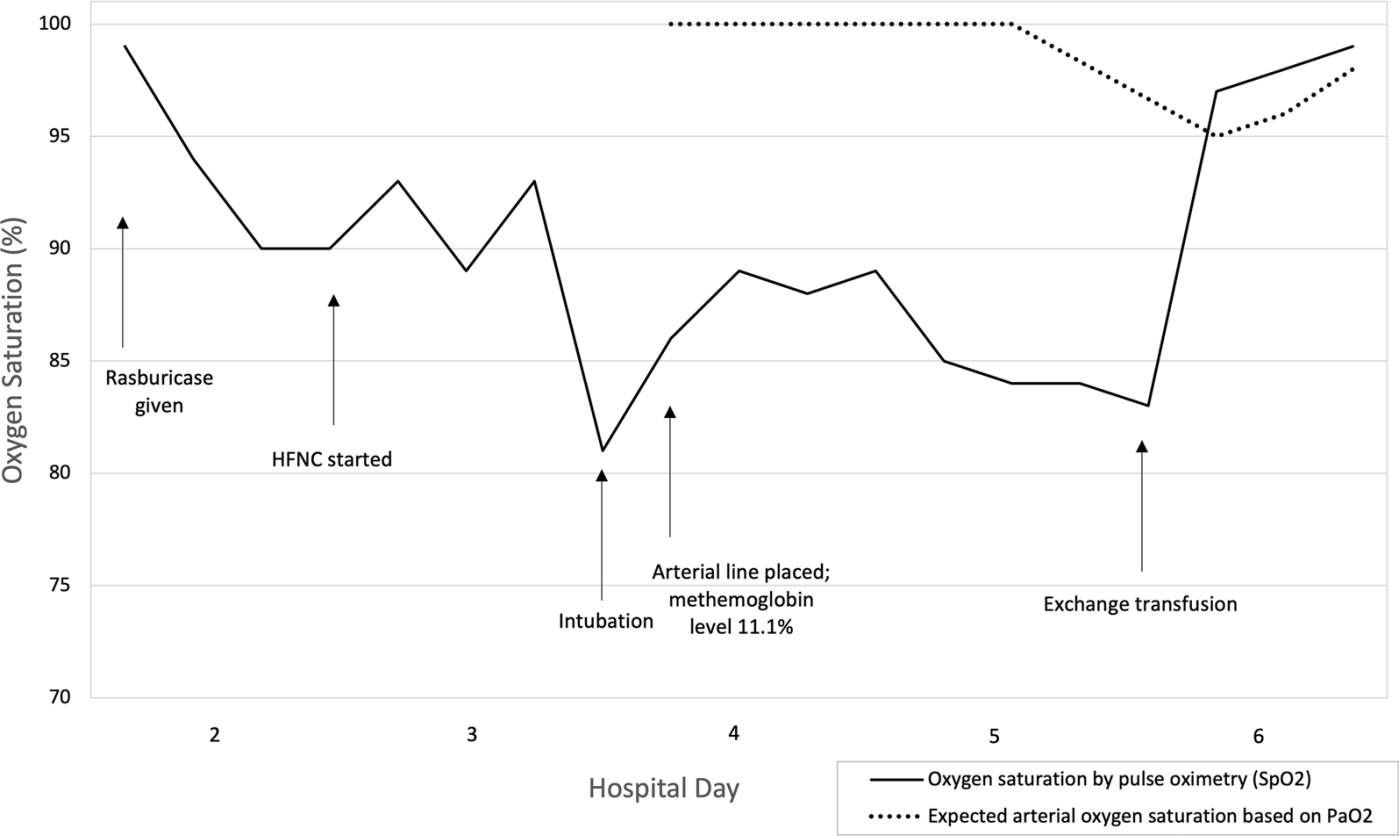
Changes in oxygenation saturation with rasburicase administration and subsequent treatment. The oxygen saturation by pulse oximetry began to decline following rasburicase. After intubation, arterial blood gas analysis showed PaO_2_ of 197 mmHg and SaO_2_ of 100%, despite a pulse oximetry reading of 86% saturation. The methemoglobin level was 11.1%. Exchange transfusion resulted in resolution of the oxygen saturation gap and methemoglobinemia. *HFNC* high-flow nasal cannula

Because methylene blue can trigger hemolytic crises in G6PD-deficient individuals, ascorbic acid was used to treat methemoglobinemia (1000 mg intravenously, 6 times a day). Over the next 48 hours, oxygen saturation remained low, hemolysis progressed (hematocrit 27.6%, MCV 82 fL, and haptoglobin 2.35 μmol/L [normal 9.76–31.4 μmol/L]), and lactate levels rose (3.0 mmol/L [normal 0.5–2.2 mmol/L]). Single volume exchange transfusion was performed on hospital day 4, leading to rapid improvement in saturation and closure of the oxygen saturation gap. He was successfully extubated the following day. The trends in oxygen saturation by pulse oximetry and expected saturation values (according to PaO_2_) are shown in Fig. 2.

He received additional packed red blood cell transfusions for ongoing anemia associated with hemolysis, which resolved by hospital day 10. Hemodialysis was discontinued on day 16, and he was discharged home on day 20. Renal function was normal when checked at 10 days and at 3 months after discharge. There were no further episodes of rhabdomyolysis. Genetic testing with a panel of genes associated with rhabdomyolysis [10] confirmed presence of the “A-” G6PD variant (c.[202G>A; 376A>G] p.[Val68Met; Asn126Asp]) and a heterozygous variant of unknown significance in the dysferlin gene (DYSF c.4510G>A p.Val1504Ile).

## Discussion

Although > 7% of the world’s population is affected with G6PD deficiency [11], rhabdomyolysis in G6PD deficiency has rarely been reported [7, 12]. One possible explanation is that skeletal muscle has other enzymes, such as catalase and superoxide dismutase, that can remove oxidative radicals [13]. Historically, the first report of rhabdomyolysis in G6PD deficiency described a 30-year-old athlete who became comatose toward the end of a 12-km run and was found to have severe rhabdomyolysis with creatine kinase of 41 × 10^6^ U/L [14].

### Rhabdomyolysis and COVID-19

Over 50 reports describe rhabdomyolysis in COVID-19. Such patients commonly present with acute hypoxemic respiratory failure, and rhabdomyolysis and acute kidney injury develop later [15]. Alternatively, severe rhabdomyolysis may be the main clinical feature with only mild (or absent) COVID-19 respiratory symptoms [16], as in our patient. None of the reports described investigation for predispositions to rhabdomyolysis.

### G6PD deficiency and coronavirus

Clinical observations suggest that G6PD deficiency may worsen outcomes following SARS-CoV-2 infection. Hospitalized patients with G6PD deficiency and COVID-19 pneumonia had lower PaO_2_/FiO_2_ ratio, longer duration of mechanical ventilation, and reduced hemoglobin level compared with those with normal G6PD [17].

Higher mortality rates have also been observed among African American males admitted for COVID-19 pneumonia [18, 19]. Potential explanations for such outcomes include the higher frequency of G6PD deficiency in this population, in addition to socioeconomic factors [17, 20, 21].

Molecular mechanisms for the interaction between G6PD deficiency and SARS-CoV-2 have not yet been identified, but various classes of viruses can increase oxidative stress in the host [22]. Furthermore, *in vitro* infection of G6PD-deficient fibroblasts with human coronavirus 229E yielded nearly 12-fold higher viral gene expression, 3-fold higher viral protein production, and reduced cell viability, compared with results with normal fibroblasts [23]. Conceivably, G6PD-deficient cells may be more susceptible to oxidative stress from SARS-CoV-2.

### G6PD deficiency and methemoglobinemia

Our patient had persistently low oxygen saturation with discordant PaO_2_ level due to methemoglobinemia after one dose of rasburicase for hyperuricemia. Methemoglobinemia is a rare complication of G6PD deficiency following use of rasburicase. In converting uric acid to allantoin, rasburicase produces hydrogen peroxide, which causes oxidative damage in G6PD-deficient red blood cells [24]. Methylene blue is typically the first-line treatment for methemoglobinemia, but it requires reduction to leucomethylene blue by NADPH. Therefore, it is avoided in G6PD deficiency [25]. Other treatments include ascorbic acid and exchange transfusion [26].

## Conclusions

Our case shows a dramatic and unexpected presentation of G6PD deficiency, with rhabdomyolysis and renal failure, in a young man with SARS-CoV-2 infection. Early screening for G6PD deficiency in patients with rhabdomyolysis can help direct treatment and prevent complications. Screening may be especially useful for individuals of African or Middle Eastern descent, among whom the prevalence of G6PD deficiency is higher.

## Data Availability

Not applicable.

## Abbreviations

G6PD: Glucose-6-phosphate dehydrogenase
NADPH: Nicotinamide adenine dinucleotide phosphate
AST: Aspartate aminotransferase
ALT: Alanine aminotransferase
PCR: Polymerase chain reaction
HFNC: High-flow nasal cannula
MCV: Mean corpuscular volume
PaO_2_: Partial pressure of oxygen
FiO_2_: Fraction of inspired oxygen
